# Unraveling the treatment effects of huanglian jiedu decoction on drug-induced liver injury based on network pharmacology, molecular docking and experimental validation

**DOI:** 10.1186/s12906-024-04517-y

**Published:** 2024-06-07

**Authors:** Yaochen Xie, Shuchen Gong, Lingkun Wang, Zhaoxu Yang, Chen Yang, Guilin Li, Huiyan Zha, Shuying Lv, Boneng Xiao, Xiaoyu Chen, Zhenning Di, Qiaojun He, Jincheng Wang, Qinjie Weng

**Affiliations:** 1 Center for Drug Safety Evaluation and Research, Zhejiang Province Key Laboratory of Anti- Cancer Drug Research, College of Pharmaceutical Sciences, Zhejiang University, Hangzhou, 310007 China; 2 Taizhou Institute of Zhejiang University, Taizhou, 318000 China; 3 Hangzhou Institute of Innovative Medicine, College of Pharmaceutical Sciences, Zhejiang University, Hangzhou, 310058 China; 4 School of Pharmacy, Nanjing University of Chinese Medicine, Nanjing, 210023 China; 5 ZJU-Xinchang Joint Innovation Center (TianMu Laboratory), Gaochuang Hi-Tech Park, Xinchang, 312500 Zhejiang China; 6 Department of Cardiology, Second Affiliated Hospital, School of Medicine, Zhejiang University, Hangzhou, 310058 Zhejiang China; 7 Beijing Life Science Academy, Beijing, 102200 China; 8 The Second Affiliated Hospital, Zhejiang University School of Medicine, Hangzhou, 310058 China

**Keywords:** Huanglian Jiedu Decoction, Network pharmacology, Drug-induced liver injury, Molecule docking, Tryptophan metabolism, Q-TOF

## Abstract

Huanglian Jiedu Decoction (HJD) is a well-known Traditional Chinese Medicine formula that has been used for liver protection in thousands of years. However, the therapeutic effects and mechanisms of HJD in treating drug-induced liver injury (DILI) remain unknown. In this study, a total of 26 genes related to both HJD and DILI were identified, which are corresponding to a total of 41 potential active compounds in HJD. KEGG analysis revealed that Tryptophan metabolism pathway is particularly important. The overlapped genes from KEGG and GO analysis indicated the significance of CYP1A1, CYP1A2, and CYP1B1. Experimental results confirmed that HJD has a protective effect on DILI through Tryptophan metabolism pathway. In addition, the active ingredients Corymbosin, and Moslosooflavone were found to have relative strong intensity in UPLC-Q-TOF-MS/MS analysis, showing interactions with CYP1A1, CYP1A2, and CYP1B1 through molecule docking. These findings could provide insights into the treatment effects of HJD on DILI.

**Supplementary Information:**

The online version contains supplementary material available at 10.1186/s12906-024-04517-y.

## Background

Drug-induced liver injury (DILI) is a common adverse reaction caused by certain drugs, which can potentially lead to liver damage and even death in severe cases [1]. Studies have shown that DILI has an occurrence rate of up to 19.1 per 100,000 people every year in some European countries, and an astonishing 23.8 per 100,000 people every year in China [2]. Drugs used for anti-bacterial, anti-tumor, and anti-inflammatory purposes have the potential to induce liver injury. Specifically, many drugs used for fever and pain relief contain Acetaminophen (APAP) as their active ingredients, which can cause acute liver failures or even death if taken in excessive amounts without supervision [3]. This is also a common chemical substance used for studying drug-induced liver injury in mice [4, 5]. Currently, the only drug approved by the U.S. Food and Drug Administration for treating DILI is N-Acetylcysteine. However, it has several limitations, including poor bioavailability, a narrow time window for effective use, and high protein binding. In addition, it could only be applied to limited situations [6]. Therefore, it is in great need of novel potential targets and treatment methods.

Traditional Chinese medicine (TCM) has been developed in Asia over thousands of years, and continues to play a critical role in daily healthcare due to its low toxicity [7], and is still utilized by a large number of individuals [8, 9]. Huanglian Jiedu decoction (HJD), which is composed of four different ingredients Huanglian, Huangbai, Huangqin and Zhizi, has a long consumption history in China and east Asia. Previous studies have elucidated that HJD has shown potential acting as enteral nutrition in septic patients [10], treating diabetes by increasing activity of intestinal pancreatic lipase [11], and intervening gastrointestinal dysfunction [12] in clinical practices. In addition to the clinical use, HJD was believed to have the ability in ameliorating inflammatory in DSS-induced colitis [13], X-ray-induced radiation dermatitis injury [14], kidney injury [15], cardio injury [16], and atopic dermatitis [17] in mice. Furthermore, it could remodel the periphery microenvironment to inhibit Alzheimer’s disease in mice [18].

Especially, HJD has also been found to have effects on liver diseases. It has already been reported that HJD could become a potential treatment for hepatocellular carcinoma by inducing apoptosis and impairing cell proliferation in both in vitro HCC cell and in vivo mouse models [19]. In addition, it could induce macrophages M2 polarization on livers of hyperlipidemia mice to protect against injury [20]. On top of that, HJD has been reported to have hepatoprotective effects against carbon tetrachloride induced acute liver injury through inhibiting triglyceride accumulation [21], and could maintain the metabolism homeostasis and relieve cholestatic liver injuryin animal models [22, 23]. HJD was also reported to have the ability in strengthening anti-inflammation and anti-oxidation to protect sepsis induced acute liver injury [24, 25]. Simultaneously, HJD was believed to have the ability in treating Metabolic Associated Fatty Liver Disease through AMPK-mTOR signaling pathway [26]. When it comes to the in vitro experiments, HJD could inhibit HepG2 cell growth to treat liver cancer [27]. These results indicates that HJD might have its function in treating DILI in the mice model, however, there is limited research in this field, and the underlying mechanisms are still unknown. This lack of knowledge hinders its clinical application and we believe that applying HJD in a mice model would be a suitable choice to study DILI.

Network pharmacology combines network biology and polypharmacology, expanding opportunities for identifying potential drug targets [28, 29] through an integrated reductionist, systems approach using combination of computational and experimental methods. Thus, it could provide a powerful tool for analyzing “network target, multi-components” in TCM through identifying active compounds and targets. By applying this approach, the mechanisms of herbal formula’s mechanisms working on biological systems can be thoroughly studied and experimental validation can further confirm the effects [30, 31]. Several studies have already been conducted on network pharmacology, and experimental validation in the field of DILI [32, 33].

In this article, we have demonstrated the potential of HJD in protecting against DILI. Our study employed a combination of network pharmacology, molecular docking, and experimental verification to identify the active compounds, pathways, and targets involved. Our findings indicate that HJD exerts its therapeutic effects on DILI through the Tryptophan metabolism pathway. The active ingredients, Corymbosin and Moslosooflavone could interact with the targets, CYP1A1, CYP1A2, and CYP1B1. Overall, our data provide a comprehensive understanding and valuable insights into the mechanisms underlying the liver protective activity of HJD.

## Results

### Drug likeness and compounds selection in HJD

Initially, according to network pharmacology study of HJD on other diseases [34–36], we used the TCMSP database to acquire potential active ingredients in HJD, discovering a total of 85 active compounds (names, origins and numbers shown in Table 1) selected from four different herbs. In cases where the same compound was present in multiple herbs, it was only counted once. Huanglian, Huangbai, Huangqin and Zhizi contained 14, 37, 36 and 15 active ingredients, respectively.

**Table 1:** Potential Active Compounds in HJD

Number	Molecule Name	Latin Name
Compound 1	Berberine	*Coptis chinensis* Franch, *Phellodendron amurense* Rupr
Compound 2	Obacunone	*Coptis chinensis* Franch, *Phellodendron amurense* Rupr
Compound 3	Berberrubine	*Coptis chinensis* Franch, *Phellodendron amurense* Rupr
Compound 4	Epiberberine	*Coptis chinensis* Franch, *Scutellaria baicalensis* Georgi
Compound 5	(R)-Canadine	*Coptis chinensis* Franch
Compound 6	Berlambine	*Coptis chinensis* Franch
Compound 7	Corchoroside A_qt	*Coptis chinensis* Franch
Compound 8	Magnograndiolide	*Coptis chinensis* Franch, *Phellodendron amurense* Rupr
Compound 9	Palmidin A	*Coptis chinensis* Franch, *Phellodendron amurense* Rupr
Compound 10	Palmatine	*Coptis chinensis* Franch, *Phellodendron amurense* Rupr
Compound 11	Quercetin	*Coptis chinensis* Franch, *Phellodendron amurense* Rupr, *Gardenia jasminoides* Ellis
Compound 12	Coptisine	*Coptis chinensis* Franch, *Phellodendron amurense* Rupr, *Scutellaria baicalensis* Georgi
Compound 13	Worenine	*Coptis chinensis* Franch, *Phellodendron amurense* Rupr
Compound 14	Moupinamide	*Coptis chinensis* Franch
Compound 15	Kihadalactone A	*Phellodendron amurense* Rupr
Compound 16	Phellavin_qt	*Phellodendron amurense* Rupr
Compound 17	Delta 7-stigmastenol	*Phellodendron amurense* Rupr
Compound 18	Phellopterin	*Phellodendron amurense* Rupr
Compound 19	Dehydrotanshinone II A	*Phellodendron amurense* Rupr
Compound 20	Delta7-Dehydrosophoramine	*Phellodendron amurense* Rupr
Compound 21	Dihydroniloticin	*Phellodendron amurense* Rupr
Compound 22	Kihadanin A	*Phellodendron amurense* Rupr
Compound 23	Niloticin	*Phellodendron amurense* Rupr
Compound 24	Rutaecarpine	*Phellodendron amurense* Rupr
Compound 25	Skimmianin	*Phellodendron amurense* Rupr
Compound 26	Chelerythrine	*Phellodendron amurense* Rupr
Compound 27	Stigmasterol	*Phellodendron amurense* Rupr, *Scutellaria baicalensis* Georgi, *Gardenia jasminoides* Ellis
Compound 28	Cavidine	*Phellodendron amurense* Rupr
Compound 29	Candletoxin A	*Phellodendron amurense* Rupr
Compound 30	Hericenone H	*Phellodendron amurense* Rupr
Compound 31	Hispidone	*Phellodendron amurense* Rupr
Compound 32	beta-sitosterol	*Phellodendron amurense* Rupr, *Scutellaria baicalensis* Georgi, *Gardenia jasminoides* Ellis
Compound 33	Fumarine	*Phellodendron amurense* Rupr
Compound 34	Isocorypalmine	*Phellodendron amurense* Rupr
Compound 35	Phellamurin_qt	*Phellodendron amurense* Rupr
Compound 36	(S)-Canadine	*Phellodendron amurense* Rupr
Compound 37	Poriferast-5-en-3beta-ol	*Phellodendron amurense* Rupr
Compound 38	Campesterol	*Phellodendron amurense* Rupr
Compound 39	Dihydroniloticin	*Phellodendron amurense* Rupr
Compound 40	Melianone	*Phellodendron amurense* Rupr
Compound 41	Phellochin	*Phellodendron amurense* Rupr
Compound 42	Thalifendine	*Phellodendron amurense* Rupr
Compound 43	Acacetin	*Scutellaria baicalensis* Georgi
Compound 44	Wogonin	*Scutellaria baicalensis* Georgi
Compound 45	(2R)-7-hydroxy-5-methoxy-2-phenylchroman-4-one	*Scutellaria baicalensis* Georgi
Compound 46	Baicalein	*Scutellaria baicalensis* Georgi
Compound 47	5,8,2’-Trihydroxy-7-methoxyflavone	*Scutellaria baicalensis* Georgi
Compound 48	5,7,2,5-tetrahydroxy-8,6-dimethoxyflavone	*Scutellaria baicalensis* Georgi
Compound 49	Carthamidin	*Scutellaria baicalensis* Georgi
Compound 50	2,6,2’,4’-tetrahydroxy-6’-methoxychaleone	*Scutellaria baicalensis* Georgi
Compound 51	(2 S)-dihydrobaicalein	*Scutellaria baicalensis* Georgi
Compound 52	Eriodyctiol (flavanone)	*Scutellaria baicalensis* Georgi
Compound 53	Salvigenin	*Scutellaria baicalensis* Georgi
Compound 54	5,2’,6’-Trihydroxy-7,8-dimethoxyflavone	*Scutellaria baicalensis* Georgi
Compound 55	5,7,2’,6’-Tetrahydroxyflavone	*Scutellaria baicalensis* Georgi
Compound 56	Dihydrooroxylin A	*Scutellaria baicalensis* Georgi
Compound 57	Skullcapflavone II	*Scutellaria baicalensis* Georgi
Compound 58	Oroxylin A	*Scutellaria baicalensis* Georgi
Compound 59	Panicolin	*Scutellaria baicalensis* Georgi
Compound 60	5,7,4’-Trihydroxy-8-methoxyflavone	*Scutellaria baicalensis* Georgi
Compound 61	Neobaicalein	*Scutellaria baicalensis* Georgi
Compound 62	Dihydrooroxylin	*Scutellaria baicalensis* Georgi
Compound 63	Sitosterol	*Scutellaria baicalensis* Georgi
Compound 64	Norwogonin	*Scutellaria baicalensis* Georgi
Compound 65	5,2’-Dihydroxy-6,7,8-trimethoxyflavone	*Scutellaria baicalensis* Georgi
Compound 66	ent-Epicatechin	*Scutellaria baicalensis* Georgi
Compound 67	bis[(2 S)-2-ethylhexyl] benzene-1,2-dicarboxylate	*Scutellaria baicalensis* Georgi
Compound 68	Supraene	*Scutellaria baicalensis* Georgi, *Gardenia jasminoides* Ellis
Compound 69	Diisooctyl phthalate	*Scutellaria baicalensis* Georgi
Compound 70	Moslosooflavone	*Scutellaria baicalensis* Georgi
Compound 71	11,13-Eicosadienoic acid, methyl ester	*Scutellaria baicalensis* Georgi
Compound 72	5,7,4’-trihydroxy-6-methoxyflavanone	*Scutellaria baicalensis* Georgi
Compound 73	5,7,4’-trihydroxy-8-methoxyflavanone	*Scutellaria baicalensis* Georgi
Compound 74	Rivularin	*Scutellaria baicalensis* Georgi
Compound 75	Crocetin	*Gardenia jasminoides* Ellis
Compound 76	3-Epioleanolic acid	*Gardenia jasminoides* Ellis
Compound 77	Ammidin	*Gardenia jasminoides* Ellis
Compound 78	Sudan III	*Gardenia jasminoides* Ellis
Compound 79	Kaempferol	*Gardenia jasminoides* Ellis
Compound 80	Mandenol	*Gardenia jasminoides* Ellis
Compound 81	Isoimperatorin	*Gardenia jasminoides* Ellis
Compound 82	Ethyl oleate (NF)	*Gardenia jasminoides* Ellis
Compound 83	Corymbosin	*Gardenia jasminoides* Ellis
Compound 84	3-Methylkempferol	*Gardenia jasminoides* Ellis
Compound 85	Genipin 1-gentiobioside	*Gardenia jasminoides* Ellis

### Targets prediction of HJD in treating DILI

**Fig. 1:**
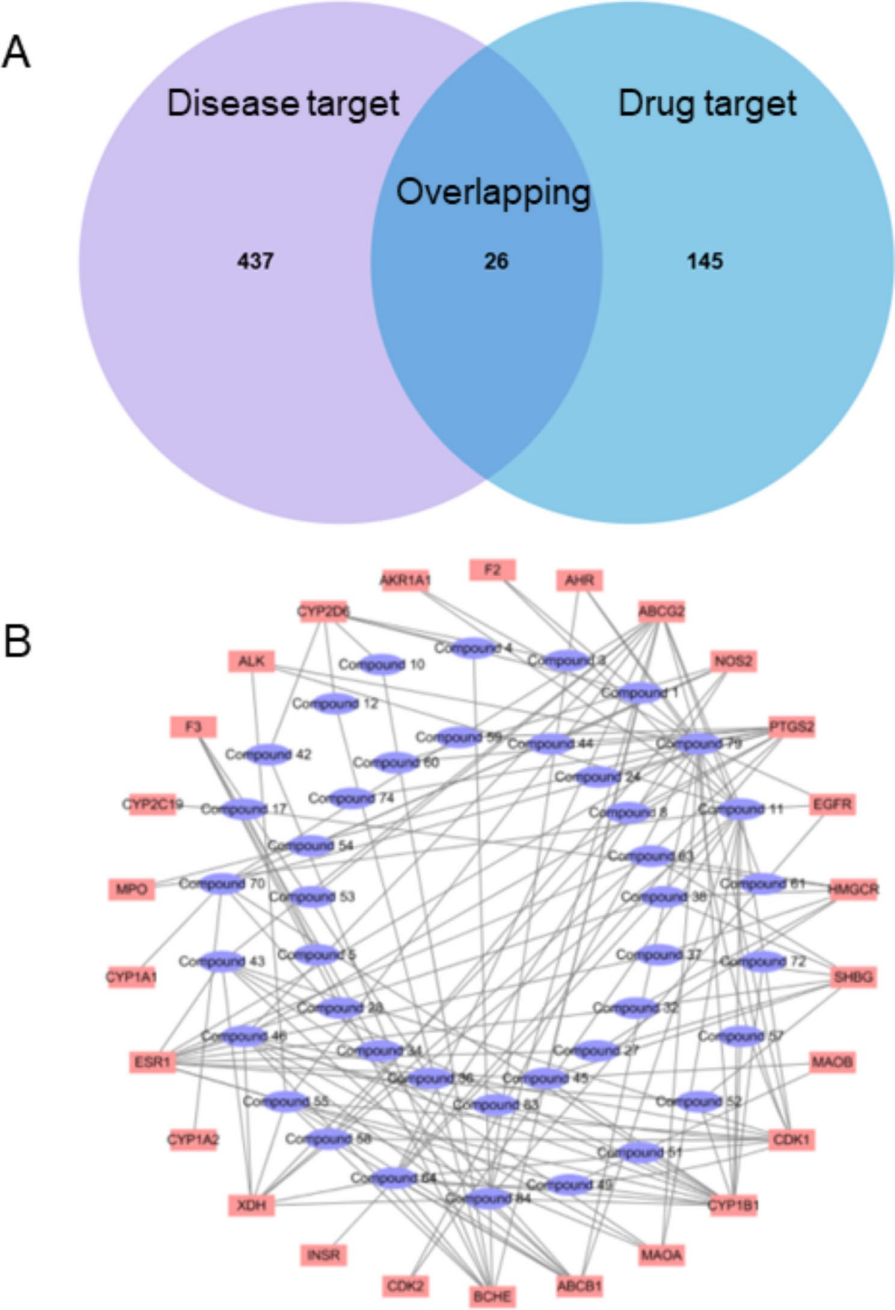
The relationship between DILI targets and active ingredients in HJD. (**A**) The Venn diagram of drug targets, disease targets and their overlapping during network construction process of HJD and DILI. (**B**) The network between the overlapped targets and their related active compounds in HJD

After identifying all the potential ingredients, the main concern was determining which targets they could interact with. These compounds showed interactions with a total of 171 potential target genes. From the database, a total of 463 genes related to DILI were selected. By comparing these two gene sets, we identified 26 potential targets of HJD for the treatment of DILI (Fig. 1A), which associated with a total of 41 potential active compounds in HJD. Based on these relationships, a network was then constructed (Fig. 1B). Within this network, the most important three potential active compounds are Compound 79, Compound 11 and Compound 46 which had 16, 15 and 10 degrees, respectively. Furthermore, the most significant three potential targets were CYP1B1, ESR1 and CDK1, which have 15, 12 and 11 degrees, respectively.

**Fig. 2:**
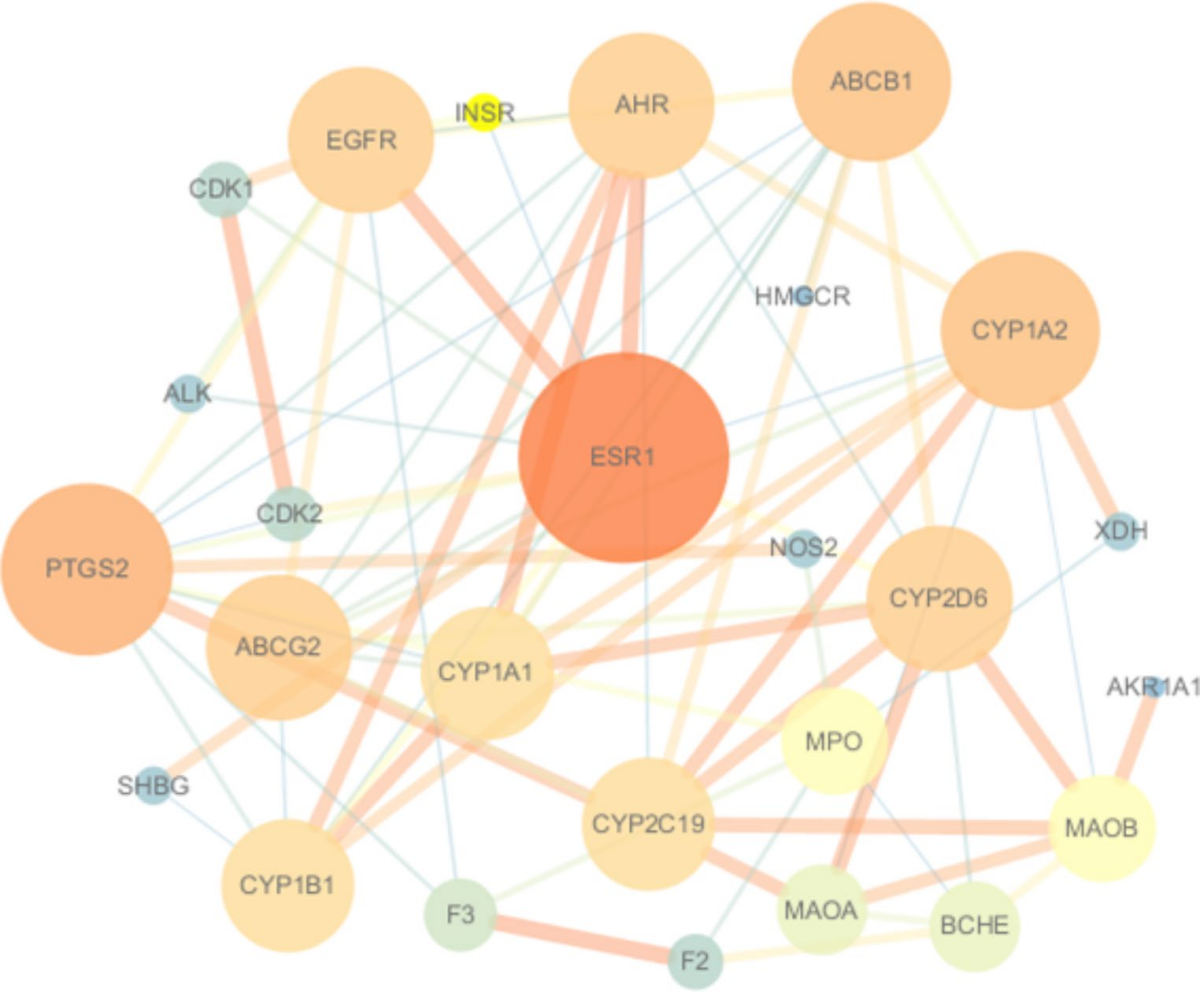
PPI network of the overlapped 26 potential targets that could be used in HJD for treating DILI

In order to identify potential targets for treating DILI, a total of 26 overlapped genes were analyzed. The results revealed that these genes formed a network with 76 edges and each node had an average degree of 5.85. The PPI enrichment p-value was found to be lower than 1 × 10^–16^. Based on network analyzer on Cytoscape (Fig. 2), the core targets played critical roles including ESR1, PTGS2, CYP1A2, ABCB1, AHR, CYP2D6, EGFR and ABCG2.

### Network pharmacology and in vitro experiments elucidate that HJD protects against DILI through Tryptophan metabolism pathway

**Fig. 3:**
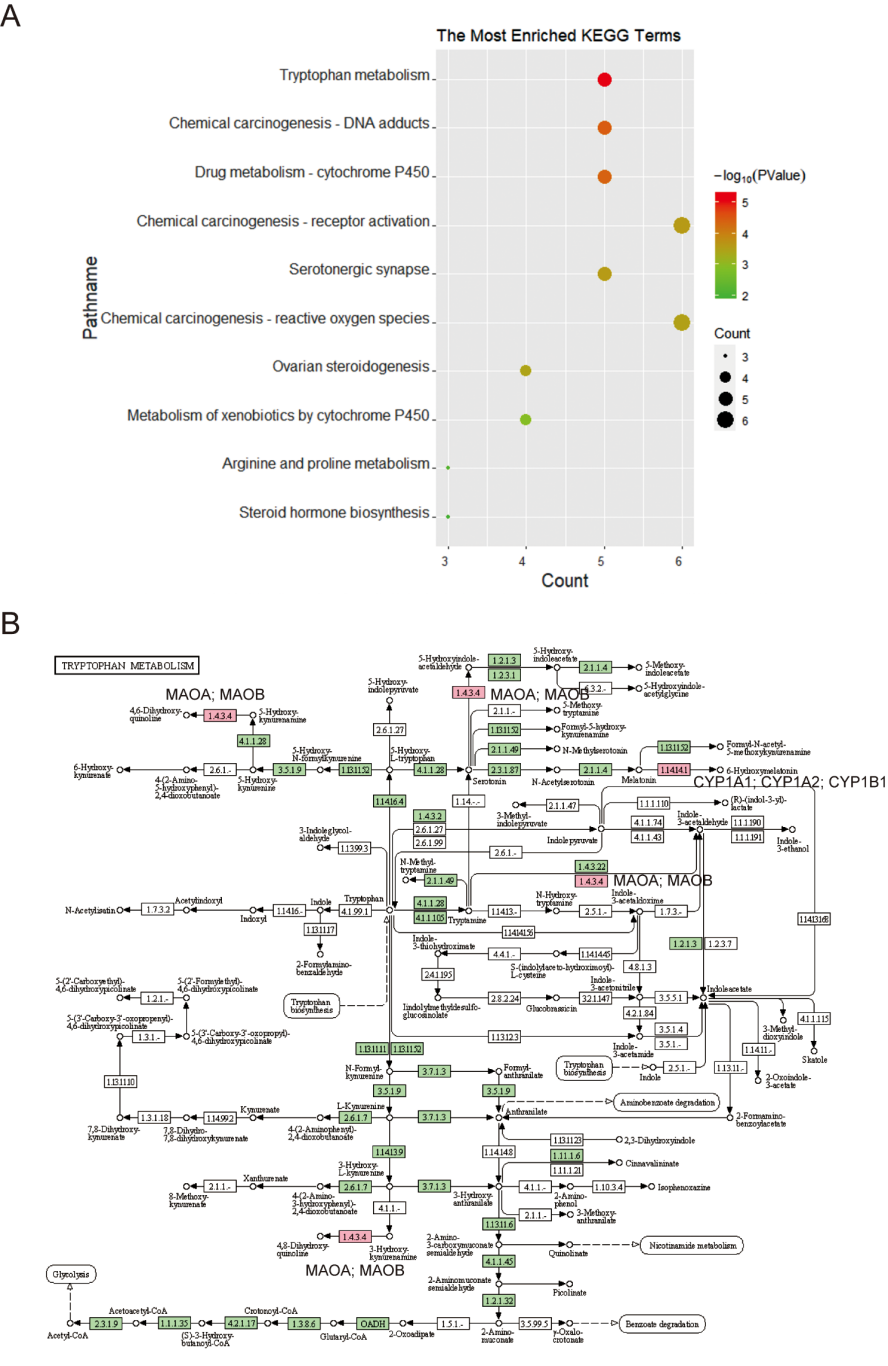
Pathway analysis of the protective effects HJD against DILI. (**A**) The most enriched pathways for KEGG; (**B**) The signaling transduction pathway of Tryptophan metabolism signaling pathway

KEGG analysis was conducted using the results obtained from the PPI network. The results revealed a total of 26 enriched pathways, where the most significant ones were Tryptophan metabolism, Chemical carcinogenesis - DNA adducts, and Drug metabolism - cytochrome P450 (Fig. 3A). Among these pathways, the Tryptophan metabolism pathway was particularly relevant to the potential treatment of DILI, which involves CYP1A1, CYP1A2, CYP1B1, MAOA, and MAOB (Fig. 3B).

**Fig. 4:**
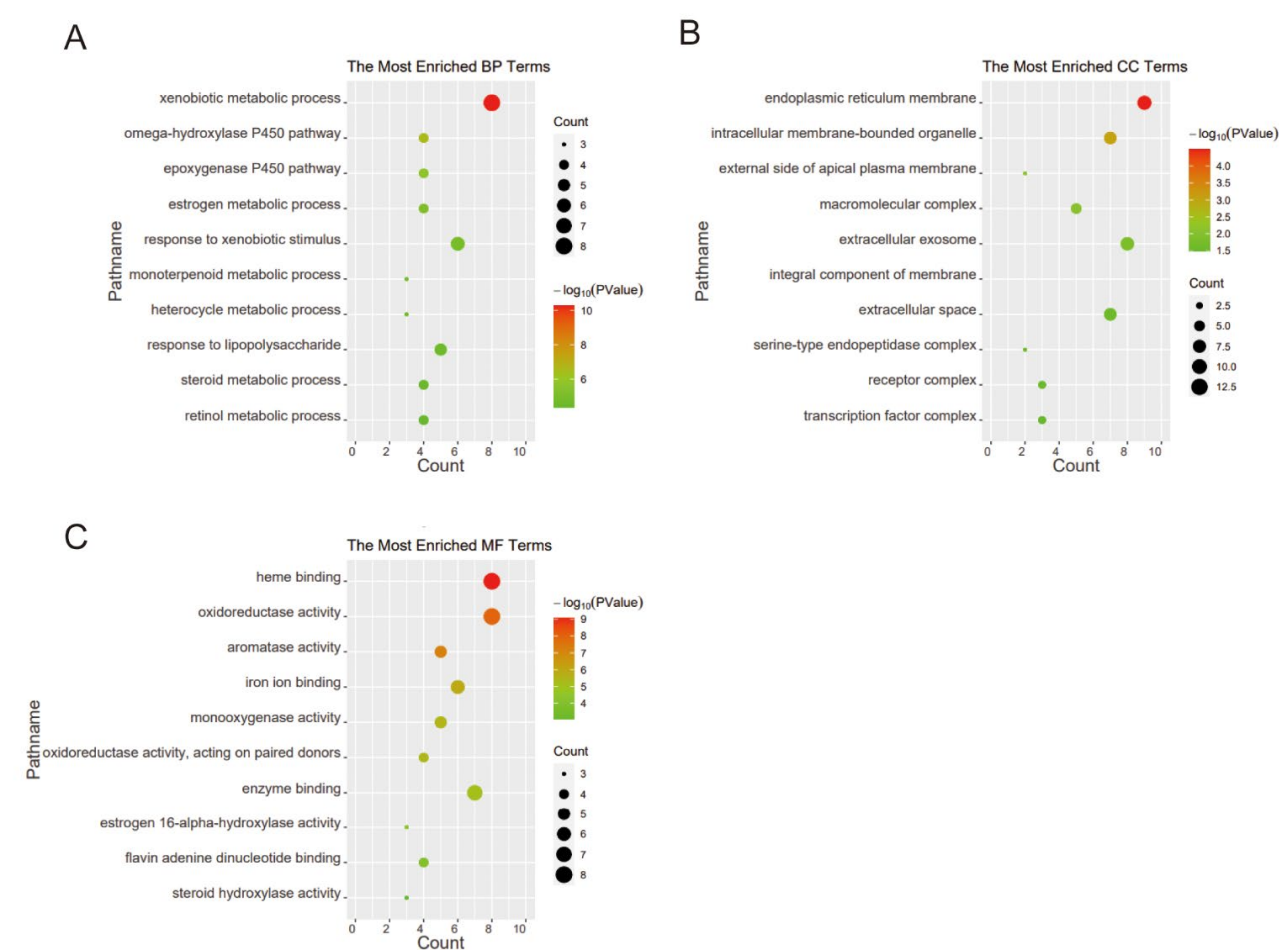
GO enrichment analysis for the top 10 most significant terms in BP (**A**), CC (**B**), and MF (**C**)

Furthermore, GO analysis was carried out, and the top significantly enriched terms were shown in Fig. 4. The Biological Process (BP) results were presented in Fig. 4A, with the most significant term being xenobiotic metabolic process. The genes participated in this process included CYP2C19, CYP1B1, CYP1A2, CYP1A1, ABCB1, CYP2D6, BCHE, and AHR. The Cellular Component (CC) results indicated that the critical targets were mainly distributed at the endoplasmic reticulum membrane, which contained CYP2C19, CYP1B1, CYP1A2, CYP1A1, CDK1, CYP2D6, HMGCR, PTGS2, and EGFR genes (Fig. 4B). The Molecular Function (MF) results revealed that these genes have functions such as heme binding, oxidoreductase activity, aromatase activity, iron ion binding, and monooxygenase activity, where the proteins enriched in heme binding included CYP2C19, CYP1B1, CYP1A2, CYP1A1, CYP2D6, MPO, PTGS2, and NOS2 (Fig. 4C).

**Fig. 5:**
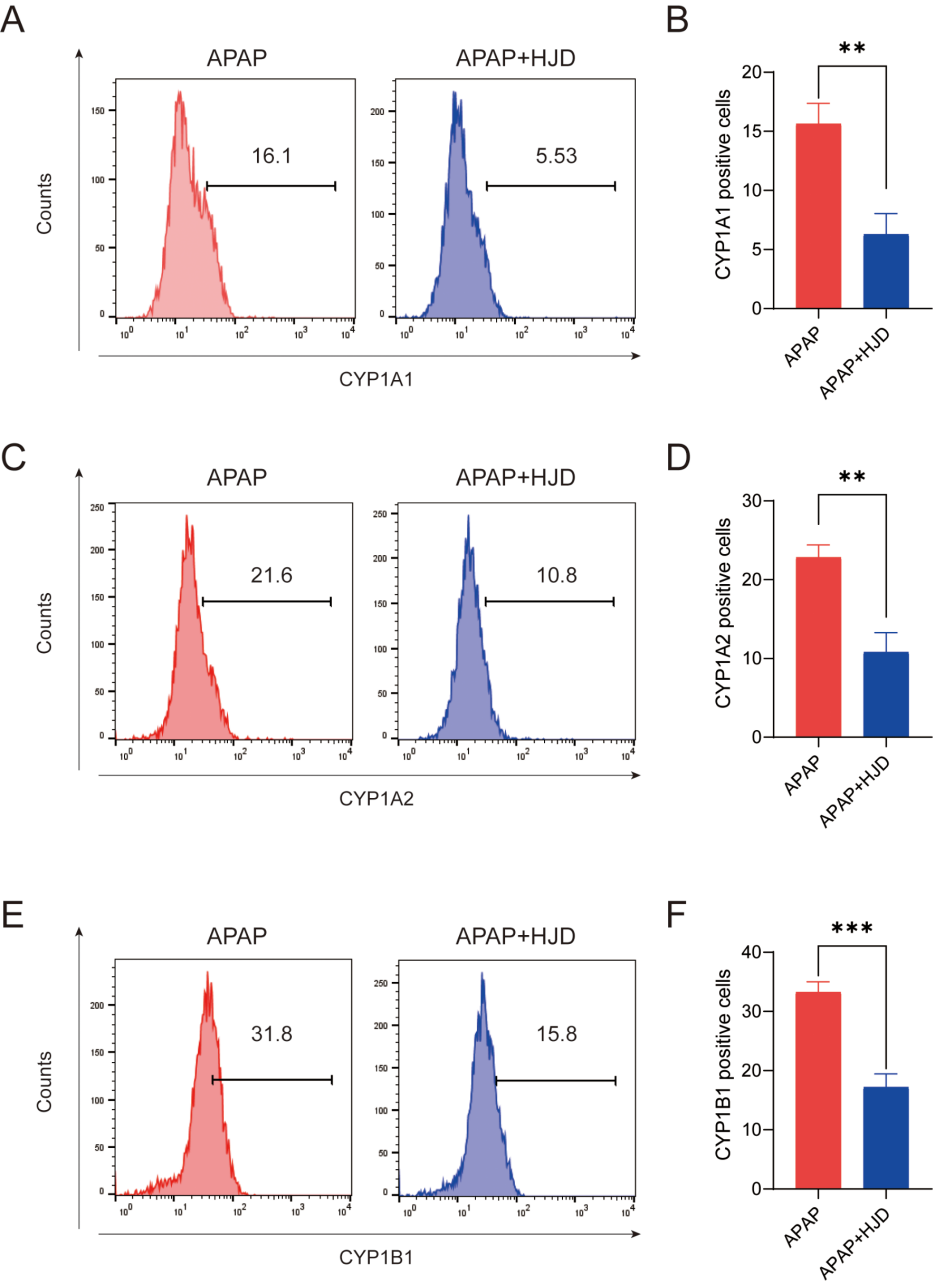
Flow cytometry analysis for CYP1A1 (**A**-**B**), CYP1A2 (**C**-**D**), and CYP1B1 (**E**-**F**). Data are presented as **, *p* < 0.01; and ***, *p* < 0.001

Through the KEGG and GO analysis, the key targets CYP1A1, CYP1A2, and CYP1B1 were overlapped, suggesting their significance. Under this circumstance, we further used flow cytometry to determine the expression levels of these proteins, the results showed that HJD could dwindle the CYP1A1, CYP1A2, and CYP1B1 positive cell levels after APAP treatment (Fig. 5A-F). Therefore, it was possible that the active ingredients in HJD docked with these targets could provide potential treatment methods for DILI.

### HJD alleviates DILI induced by APAP via Tryptophan metabolism pathway in mice

**Fig. 6:**
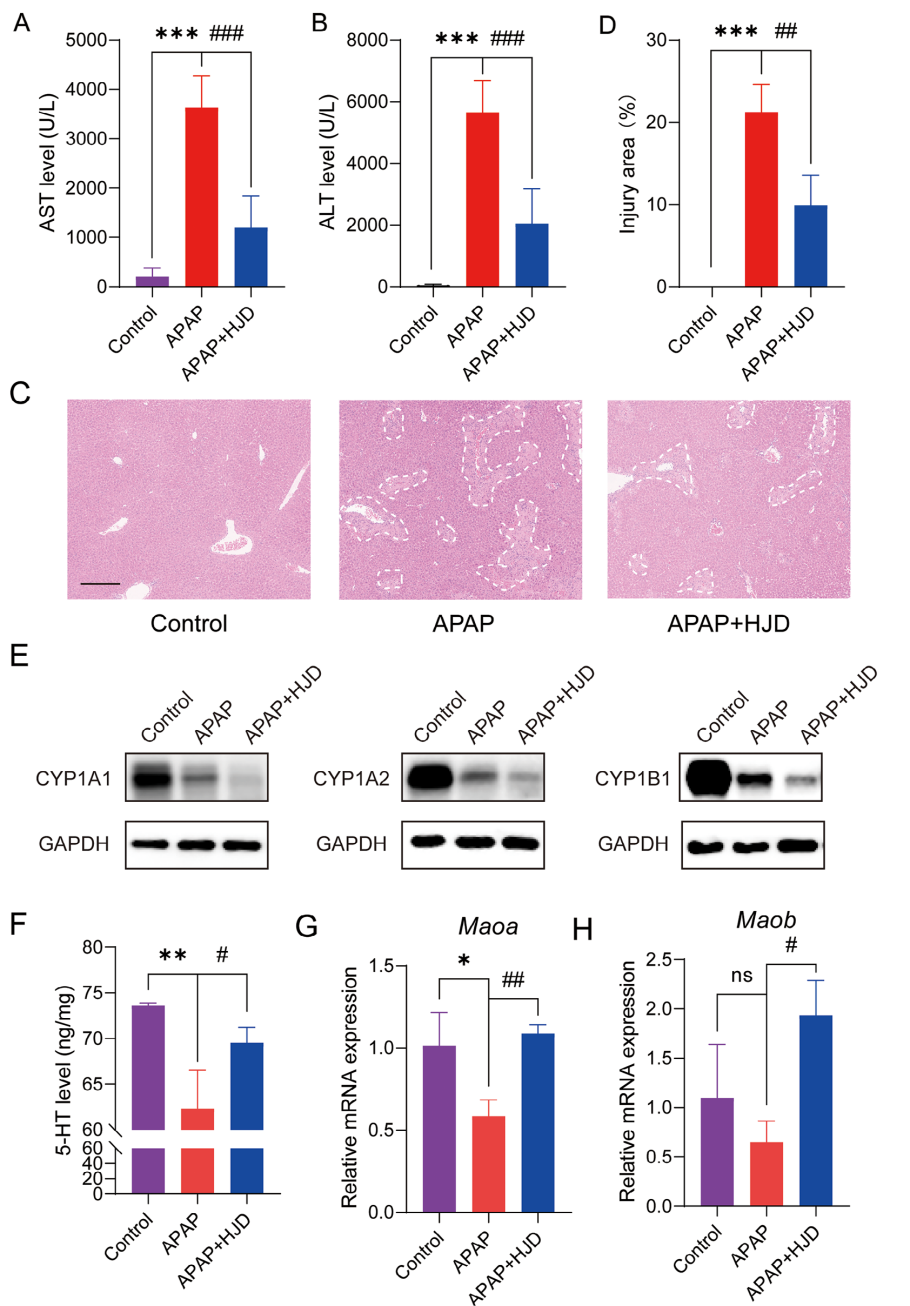
Experimental validations of HJD treatment against DILI with three identical groups (Control, APAP, and APAP + HJD). (**A**) and (**B**) ALT and AST levels (*n* = 6 animals/group). (**C**) Representative H&E staining of liver where circled place indicates places of injury, scale bar: 300 μm. (**D**) Statistics of liver injury area (*n* = 3 animals/group); (**E**) Representative western blotting analysis of liver CYP1A1, CYP1A2, and CYP1B1 expression levels in mice liver (*n* = 3 animals/group), Original images of blots are shown in Fig. S1.; (**F**) Liver 5-HT level (*n* = 3 animals/group); (**G**-**H**) qRT-PCR analysis of *Maoa* and *Maob* in mice liver (*n* = 3 animals/group). Data are presented as ns, *p* > 0.05; *, *p* < 0.05; **,  *p < *0.01; ***, *p* < 0.001 compared with Control group; ^#^, *p* < 0.05; ^##^, *p* < 0.01; ^###^, *p* < 0.001 compared with APAP group

To investigate the potential of HJD in alleviating drug-induced liver injury in vivo, mice were administered HJD for 5 consecutive days prior to APAP treatment. After 24 h of APAP treatment, liver and serum samples were collected for biochemical analysis. After receiving APAP treatment, both the levels of Alanine aminotransferase (ALT), and aspartate aminotransferase (AST) which used as a symbol for liver injury level, were increased dramatically. ALT and AST level in those received both APAP and HJD declined sharply, indicating a decrease in liver injury, compared to the group treated with APAP alone (Fig. 6A-B). Moreover, H&E staining of liver tissue showed normal cell morphology in Control group, however large area of necrosis could be witnessed for those receiving APAP treatment. In contrast, those received both APAP and HJD showed significant decrease in the area of liver necrosis (Fig. 6C-D). Since we have proven that HJD could dwindle the expression levels of CYP1A1, CYP1A2, and CYP1B1 in vitro, we further carried out western blotting analysis on the protein expression levels of in mice liver. The results showed the expression levels of all three proteins shrank after receiving APAP treatment, and the general trend experienced a further down-regulation when receiving both APAP and HJD treatment (Fig. 6E). To further confirm whether Tryptophan metabolism pathway played a key role during HJD treatment, we first carried out ELISA analysis on the metabolites of Tryptophan 5-HT in the liver tissue. The results revealed that it was downregulated in the mice liver after APAP treatment, while mice that had previously received HJD showed a recovery in 5-HT levels (Fig. 6F). Furthermore, we analyzed the relative mRNA levels of *Maoa*, and *Maob*. The results showed that the expression levels of *Maoa*, and *Maob* decreased after receiving APAP treatment, while witnessed an increment after getting HJD (Fig. 6G-H). Thus, these results suggest the Tryptophan metabolism pathway participates in the treatment of HJD against APAP induced liver injury.

### Molecule docking of active ingredients with potential targets

**Fig. 7:**
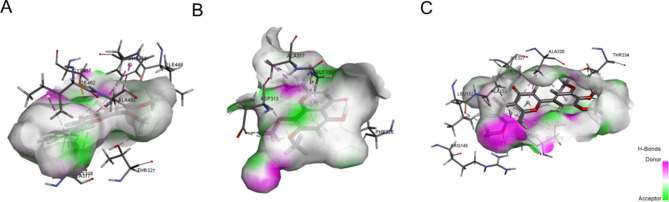
Molecule docking of active ingredients with potential targets. (**A**) The binding of CYP1A1 with Compound 70; (**B**) The binding of CYP1A2 with Compound 70; (**C**) The binding of CYP1B1 with Compound 83

To determine the major components in HJD for treating DILI, we performed docking of active ingredients related to the three key targets (Table 2). The top hit for each target was shown in Fig. 7 (A. Compound 70-CYP1A1; B. Compound 70-CYP1A2; C. Compound 83-CYP1B1).

**Table 2:** Molecule docking results of active ingredients binding with potential targets

Target	Compound	Absolute energy	Relative energy	LibDock score	Binding energy
CYP1A1	Compound 70	50.9549	0	100.611	-6.1248
CYP1A2	Compound 70	50.9549	0	111.974	765.806
CYP1B1	Compound 11	32.1631	0.007029	119.602	-85.3186
	Compound 43	36.7488	0.077824	120.34	2.6421
	Compound 45	39.5267	3.63321	107.425	31.2658
	Compound 46	37.6624	0	115.834	244.035
	Compound 49	37.8979	3.63027	120.769	-59.8867
	Compound 51	37.2207	3.66012	117.353	-45.7338
	Compound 52	34.0901	0.482728	121.715	175.814
	Compound 55	39.6749	0	116.219	148.946
	Compound 61	99.6215	19.0149	111.906	12.7113
	Compound 57	81.7051	0.525727	108.283	76,791
	Compound 64	33.4761	0	113.532	340.748
	Compound 70	50.9549	0	105.536	-14.4267
	Compound 72	40.5284	0.402813	111.821	38.0565
	Compound 79	34.4177	0	118.108	161.618
	Compound 83	96.0745	18.6841	128.209	-32.612
	Compound 84	47.7035	0	118.865	118.358

The results showed that Compound 70 docking with CYP1A1, a Pi-Pi Stacked and Amide-Pi stacked could be formed with Ala 317 and Phe 450 in Chain A. Pi-Alkyl relations were also observed between Ile 462 and Ala 463 in Chain A with Compound 70. Furthermore, a Pi-Sulfur interaction was found with Cys 457 in Chain A. Carbon Hydrogen Bond interactions were viewed at Thr 321 and Ile 449, while van der Waals interaction at Gly 318 could also influence the binding. For Compound 70 docking with CYP1A2, two Pi-alkyl interactions were witnessed at Ala 317, and Leu 497 in Chain A respectively. On top of that, a carbon hydrogen bond was shaped between the compound and Asp 313, and a Pi-Pi Stacked and Amide-Pi Stacked interactions were also observed. Indicating a diverse trend, Compound 83 docked with CYP1B1 could form a conventional hydrogen bond with Arg 145 in Chain A. For Pi-alkyl, the interactions could be found in Ala 133, Ala 330, and Cys 470 in Chain A. In addition, a Pi-Sigma interaction was observed for Ile 471 in Chain A combined three carbon-hydrogen bonds with Leu 132, Ile 327, and Thr 334 in Chain A.

### Confirmation of key ingredients in HJD

**Fig. 8:**
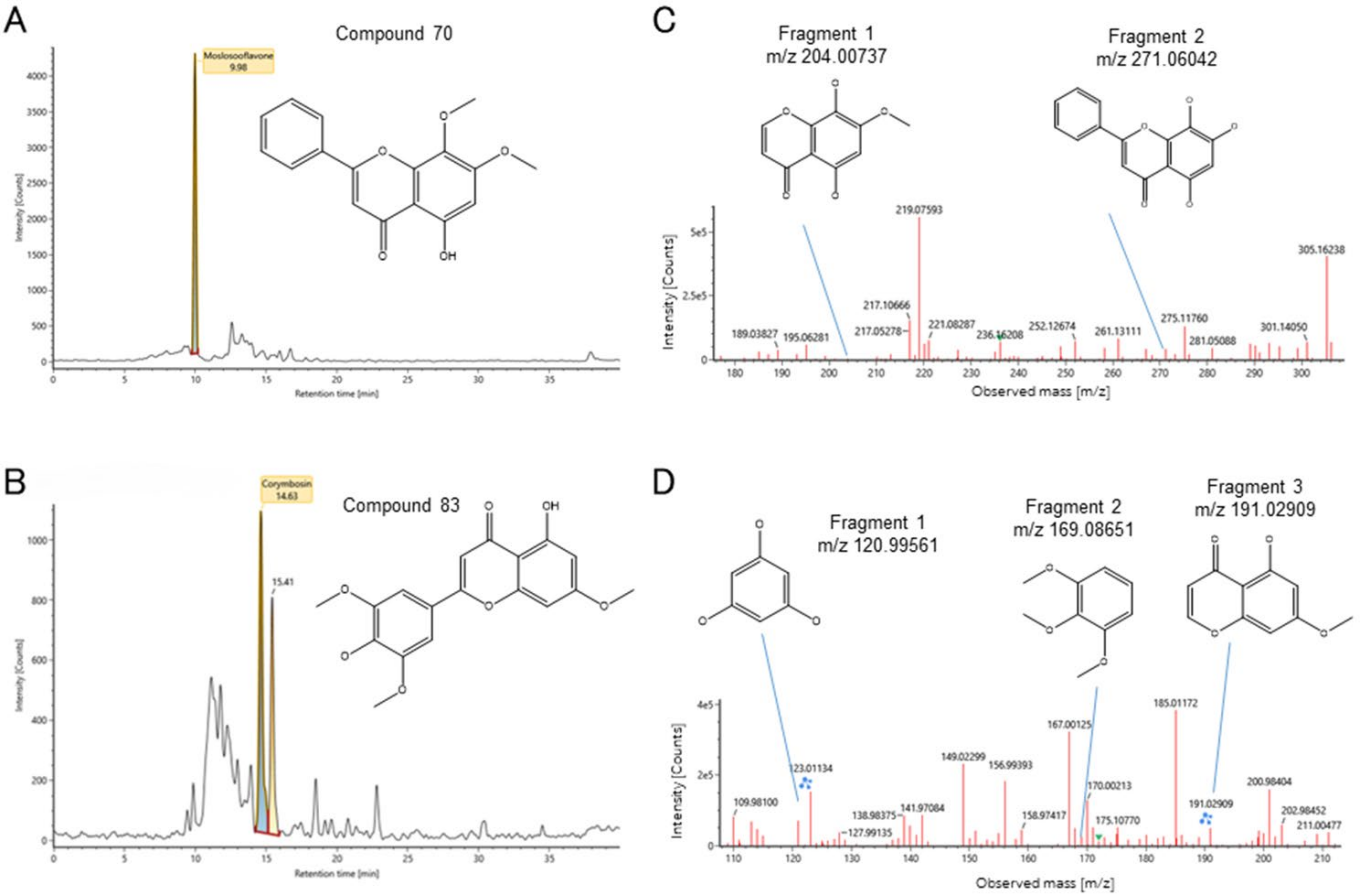
UPLC-Q-TOF-MS/MS analysis of active ingredients in HJD. (**A**) and (**B**) The intensity of Compound 70 and 83, and its retention time in UPLC-Q-TOF-MS/MS analysis; (**C**) and (**D**) Corresponding secondary mass spectra of Compound 70 and 83

The HJD decoction was analyzed to determine the presence and intensity of its two key active ingredients. Initially, we used UPLC-Q-TOF-MS/MS to detect whether Compound 70 and 83 appeared in this decoction. Afterwards, we uploaded the spectra and the chemical structure of these two compounds into UNIFI software, in which the secondary MS scan for the fragments could be used to conjecture the structure. The results showed that the retention times for Compound 70 and 83 were found to be 9.98 min, and 14.63 min, respectively (Fig. 8A and B) with relative strong intensities. Furthermore, we utilized the secondary MS scan and found two intensities which might be the fragments of Compound 70, while three intensities which might be the fragments of Compound 83 according to the molecular weight using UNIFI software (Fig. 8C and D).

## Discussion

**Fig. 9:**
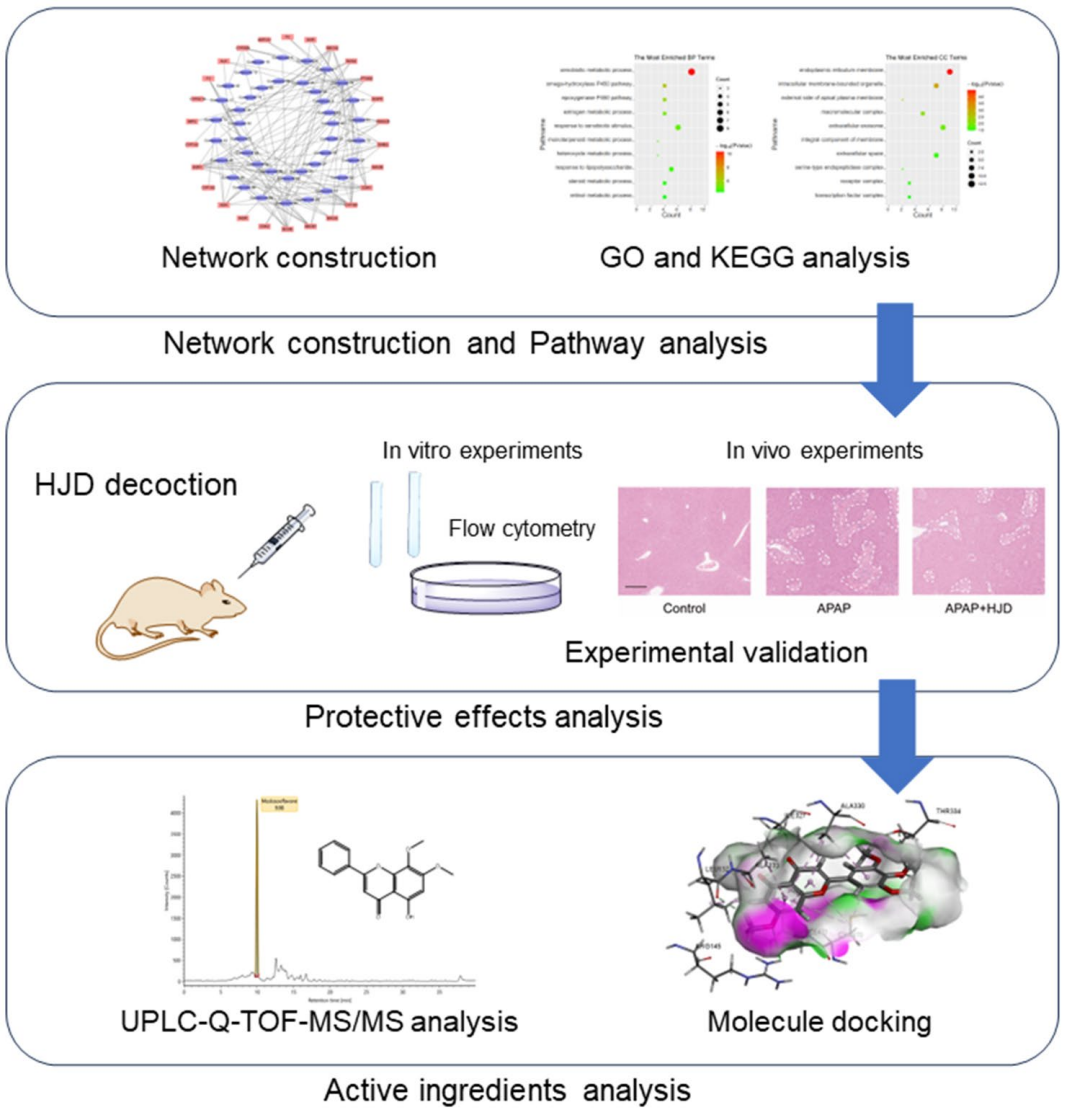
Graphical abstract of HJD treatment effect against APAP-induced liver injury

DILI is a severe and potentially life-threatening disease. TCM has shown promising potential in relieving this disease, thus we used the widely-recognized TCMSP database to find potential active ingredients in HJD (Fig. 9). In our research, we utilized PPI analysis to identify pathways involved in the biological processes of interest [37]. We analyzed the overlapped targets to determine their significance and potential interactions in DILI. According to KEGG analysis, the Tryptophan metabolism pathway emerged as the most critical pathway related to DILI, with five targets participating in this pathway. Previous reports suggest that this pathway could be influenced by the gut-liver axis and further have potential for the treatment of DILI [38–40]. Furthermore, GO analysis was carried out to identify critical targets. By overlapping the targets enriched in the Tryptophan metabolism pathway, we found that CYP1A1, CYP1A2, as well as CYP1B1 are crucial, which were subsequently used for molecule docking. Recently, there are some studies suggesting that CYP1A1 controls the nuclear translocation of aryl hydrocarbon receptor, thereby influencing DILI [41]. In addition, CYP1A2 has been implicated in exacerbating DILI [42, 43]. It has also been reported that CYP1B1 is involved in the AhR-CYP1B1-Nrf2-Keap1 pathway, which could modulate DILI [44]. Our experiments results showed that the expression levels of CYP1A1, CYP1A2, and CYP1B1 dwindled after receiving HJD treatment. Therefore, CYP1A1, CYP1A2, as well as CYP1B1 could be the potential targets in HJD treating of DILI.

Furthermore, animal experiments, whose dosage giving to mice mimics human’s daily intake and according to body surface areas, have confirmed that HJD exhibits a protective effect against DILI. It reduces the levels of ALT and AST in serum and decreases the necrosis area of liver induced by APAP. This is the first time that HJD was reported to have potentials for protecting against DILI. Mechanismly, HJD treatment is confirmed to regulate the Tryptophan metabolism pathway, as evidenced by the recovery of Tryptophan metabolites, such as 5-HT, after receiving HJD treatment.

In this study, molecular docking was performed to investigate the interaction between the above potential targets (CYP1A1, CYP1A2, and CYP1B1) and active ingredients in HJD. The compounds that achieved the highest scores in docking with each target were Corymbosin (Compound 83), and Moslosooflavone (Compound 70). Corymbosin, a glucoside isolated from Ballota glandulosissima [45], was investigated for its potential to ameliorate apoptosis process of endothelial cells [46]. Moslosooflavone was reported to have anti-oxidant activity and anti-inflammatory activity, exhibiting the potential for treating DILI [47, 48]. As expected, both of these two ingredients have relative strong intensity in UPLC-Q-TOF-MS/MS analysis.

Overall, this study presents the first report on treatment potential of HJD against DILI and explores its potential pathways. The findings of our research could contribute to the development of novel drug compounds derived from HJD for treating DILI and provide valuable insights for clinical treatment.

## Materials and methods

### Construction of HJD chemical database

The chemical compound of each component in HJD was collected from Traditional Chinese Medicine Database and Analysis Platform (TCMSP) (https://tcmsp-e.com/) [49]. In order to select compounds having potential as drug candidates, oral bioavailability (OB) and drug-likeness (DL) were applied as an indication for ADME where OB indicates the convergence of the ADME process while DL could help optimize pharmacokinetic and pharmaceutical properties [50]. In this case, only the compounds have their oral OB equal to or greater than 30% and DL equal to or greater than 0.18 were selected.

### HJD related targets screening

The corresponding protein targets were obtained from Swiss Target Prediction [51, 52] by uploading the chemical structures of each component and select Homo sapiens. In order to select targets that have better interactions with compounds, only those with a Probability score higher than or equal to 0.15 have been selected.

### Construction of DILI related genes database

In order to obtain potential genes related to DILI, GeneCards^®^: The Human Gene Database (https://www.genecards.org/) was used [53]. The targets related to DILI were then selected under the guide that the Relevance score should be no less than 1, which could give out a reasonable amount of genes suitable for analysis.

### Network construction and target overlap

The network between DILI related genes and the genes of HJD related targets were constructed using Cytoscape (https://cytoscape.org/), which could provide biomolecular interaction networks for integrated models [54]. The genes related to drugs and diseases were overlapped to get potential targets of HJD treating DILI using EVenn (http://www.ehbio.com/test/venn/), a powerful tool for generating Venn diagram [55].

### Network and key nodes analysis

After acquiring the overlapped targets, the related compounds were acquired. The network between the potential targets and the potential active compounds were constructed through Cytoscape where the network’s topological characters were obtained through ‘Network Analysis’ in Cytoscape to identify the most potential targets and compounds.

### Construction of PPI network

STRING 11.5 (https://string-db.org/) was utilized as a network database for the analysis of Protein-Protein Interactions (PPI). The overlapped genes were uploaded and analyzed using multiple protein functions and the species were set up as Homo sapiens and minimum required interaction score was set up to Median confidence (0.400). The sources of the interactions come including text mining, experiments, databases, coexpression, neighborhood, gene fusion and cooccurrence. The network was then analyzed via Cytoscape.

### KEGG pathway enrichment analysis

For further learning the pathways related to the genes, Kyoto Encyclopedia of Genes and Genomes (KEGG) (https://www.genome.jp/kegg/) was used for providing information and analysis for gene functions and linking genomic information [56]. Using KEGG Mapper tool, the related signal pathway of the genes was found and analyzed.

### Gene ontology enrichment analysis

Gene ontology (GO) analysis was carried out in order to know the biological functions of these targets on the website Database for Annotation, Visualization and Integrated Discovery (DAVID) v6.8 (https://david.ncifcrf.gov/), which could provide enriched biological themes. The overlapped genes were uploaded to the website as a gene list and selected as Homo sapiens. In Functional Annotation Clustering, three parts of the results were analyzed, including cellular components (CC), molecular functions (MF) and biological processes (BP), respectively [57].

### Molecule docking of potential ingredients

The selected ingredients were found whether they could fit in the target to have potential treatment abilities. The structures were drawn using ChemDraw 20.0 and converted to their 3D format using Chem 3D 20.0. The structure of the targets was downloaded from Protein Data Bank (https://www.rcsb.org/) for whose protein ID are 6OYU [58], 6DWM [59], and 2HI4 [60]. The targets were then modified to remove water and small binding molecules using Pymol (https://pymol.org/). The modified and prepared targets were then docked with small active ingredients in Discovery Studio 2019. The potential binding sites of the target were determined using tools in defining and editing the binding sites from PDB Site Records. LibDock in Discovery Studio 2019 was used to prepare for molecular docking where docking tolerance was set up to be 0.25, docking preference was set up as High quality.

### Preparation of HJD decoction

HJD contained four ingredients (Table 3), which are Huanglian, from dried rhizomes of *Coptis chinensis* Franch, 41.4 g; Huangqin, from dried root of *Scutellaria baicalensis* Georgi, 27.6 g; Huangbai, from dried bark of *Phellodendron amurense* Rupr, 27.6 g; and Zhizi, from dried mature fruits of *Gardenia jasminoides* Ellis, 15 g were purchased from Gushengtang and was identified by a taxonomist in the department, while the voucher samples were collected in Center for Drug Safety Evaluation and Research, Zhejiang University. The decoction was freeze dried into powder using lyophilizer (LABCONCO 6 L, USA) for 48 h. The collections were approved by Center for Drug Safety Evaluation and Research, Zhejiang University.

**Table 3:** Plant ingredients in HJD decoctions

Drug name	Plant name	Weight
Huanglian	*Coptis chinensis* Franch	41.4 g
Huangqin	*Scutellaria baicalensis* Georgi	27.6 g
Huangbai	*Phellodendron amurense* Rupr	27.6 g
Zhizi	*Gardenia jasminoides* Ellis	15 g

### Animal model

C57BL/6J mice were purchased from Zhejiang Vital River Laboratory Animal Technology Co., Ltd. and housed in specific pathogen-free environment. The treatment procedure has been approved by the Institutional Animal Care and Use Committee (IACUC) of Zhejiang University.

HJD was given at a dose of 2 g/kg dissolved in PBS (i.g) for five consecutive days, before giving 250 mg/kg APAP treatment (i.p), the mice were fastening overnight (dissolved in warm sterile saline). After 24 h, 150 mg/kg Pentobarbital Sodium was given to the mice (i.p.) to be euthanasia, whilst the blood and liver samples were collected [61]. The liver and serum sample were collected and stored at -80 °C for future analysis.

### Serum biochemistry

ALT as well as AST level in serum were measured by Cobas c 311 (Roche, Switzerland).

### Liver histopathological examination

After fixing in 10% formalin, the liver samples were then embedded in paraffin wax and sliced into 3 μm-thick sections which were used later for H&E staining. The results of staining were quantified by ImageJ (version 1.8.0).

### UPLC-Q-TOF-MS/MS analysis

The decoction powder was weighed for 1 g and dissolved in 50 mL 50% methanol, the sample was then diluted with 50% methanol for 10^7^ times and filtered through 0.22 μm membrane filter for further analysis.

UPLC analysis was carried out in a ACQUITY UPLC I-Class, where the mobile phases A (water) and B (0.1% formic acid in methanol) were set as follows: 0–3 min, 90%A; 3∼10 min, 90 − 35%A; 10–35 min, 35–5%A; 35–37 min, 5%A; 37–40 min, 90%A. 2 µL sample was injected for analysis using a ACQUITY UPLC BEH C18 (2.1 mm × 100 mm, 1.7 μm, Waters Corp.) at the temperature of 35 °C combined a flow rate at 0.3 mL/min.

The analysis was performed on a Vion IMS Qtof (Waters, USA) where positive ion mode was used for hunting potential ingredients. The desolvation gas temperature was set up to 600 L/h while the temperature was 400 °C and the capillary voltage was 2.5 kV. The collected data were processed and visualized using UNIFI v1.9.3 software.

### RNA extraction and quantitative PCR

The total RNA was extracted using Trizol (Invitrogen, CA, USA) from mice liver while cDNA was prepared by cDNA preparation supermix (TransGen Biotech, Beijing, China). Quantitative PCR was conducted utilizing SYBR Green Supermix (Bio-Rad, CA, USA) in a 20 µL reaction mixture. The reactions were executed on the Quant Studio 6 Flex Real-Time PCR System (Applied Biosystems, CA, USA). The expression levels of genes were determined by the delta-delta CT method, with normalization to *Gapdh*. The primer sequences for qPCR are detailed in Table 4.

**Table 4:** Primer sequences

Gene	Forward (5’-3’)	Reverse (5’-3’)
*mGapdh*	GGTGTGAACCATGAGAAGTATGA	GAGTCCTTCCACGATACCAAAG
*mMaoa*	GCCCAGTATCACAGGCCAC	GTCCCACATAAGCTCCACCA
*mMaob*	AACAAAAGCGATGTGATCGTGG	GCCCAACATAAGATCCTCCAAGG

### ELISA assay

The 5-HT levels in liver were measured using ELISA kit (MM-0443M1, Jiangsu Meimian Industrial Co., Ltd., China) according to the manufacturer’s protocols.

### Cell culture

AML-12 cells (Alpha Mouse Liver 12) were obtained from the Cell Bank of the Chinese Academy of Sciences (Shanghai, China), and were cultured in DMEM/F12 medium supplemented with 10% fetal bovine serum, 1% ITS Media supplement, and 40ng/mL Dexamethasone. the cells were maintained in a 37 °C, 5% CO_2_ cell humidified incubator. 100 µg/mL HJD were treated with 10 mM APAP for 24 h before flow cytometry analysis.

### Flow cytometry analysis

After the cells were fixed and permeabilized, Intracellular CYP1A1, CYP1A2, and CYP1B1 expression levels were analyzed using flow cytometry (BD FACS Canto II, BD Biosciences, USA). The primary antibodies including CYP1A1 (1:500, AP7993B, Abcepta), CYP1A2 (1:500, A0062, Abclonal), and CYP1B1 (1:500, A1377, Abclonal) were incubated with the cells at room temperature for 1 h, while the fluorescence secondary antibody was conjugated (1:500, A0516, Beyotime) with the primary antibodies for another 1 h.

### Western blotting analysis

The proteins in liver tissue were extracted in RIPA buffer together with proteinase inhibitors. The samples were then subjected to SDS-PAGE electrophoresis (10% gels) and blotted onto PVDF membranes, which were blocked with 3% BSA, and incubated with primary antibodies (anti-GAPDH (1:5000, db106, DiagBio Technology); anti-CYP1A1 (1:1000, E-AB-14,029, Elabscience); CYP1A2 (1:1000, A0062, Abclonal) anti-CYP1B1 (1:1000, DF6399, Affinity)) overnight at 4 °C. The bolts were cut before hybridization with antibodies. Afterward, the membranes were incubated with secondary antibodies and enhanced chemiluminescence detection reagents were used for visualizing. The signals were detected using GE AI600 where GAPDH was considered as the loading control.

### Statistical analysis

GraphPad Prism software (version 8.0) was used for statistical data analysis, where one-way ANOVA analysis was used for comparisons and statistical significance determination. The statistical significance was considered when *p* < 0.05 (*/^#^, *p* < 0.05; **/^##^, *p* < 0.01; and ***/^###^, *p* < 0.001).

## Conclusions

Herein, we explore the therapeutic effect and potential mechanism of HJD in the treatment of DILI based on network pharmacology and experimental validation. The KEGG analysis suggests that the Tryptophan metabolism pathway may be the most related pathway in HJD treating of DILI, while GO analysis identifies the significant targets. Animal experiments were conducted to confirm the treatment effects and enriched pathway. Based on molecule docking, Corymbosin, and Moslosooflavone, which exhibit relative strong MS reactions, might have potentials interacting with the targets CYP1A1, CYP1A2, and CYP1B1. In conclusion, our findings indicate that HJD has the potential to treat DILI through its various active ingredients and multiple targets.

### Electronic supplementary material

Below is the link to the electronic supplementary material.

Supplementary Material 1

## Data Availability

The datasets used and/or analysed during the current study available from the corresponding author on reasonable request.
