# Supplementary figures and images for: Unraveling the treatment effects of huanglian jiedu decoction on drug-induced liver injury based on network pharmacology, molecular docking and experimental validation
Source: BMC Complement Med Ther. 2024 Jun 7;24:219. doi: 10.1186/s12906-024-04517-y (PMC11157734; doi:10.1186/s12906-024-04517-y)

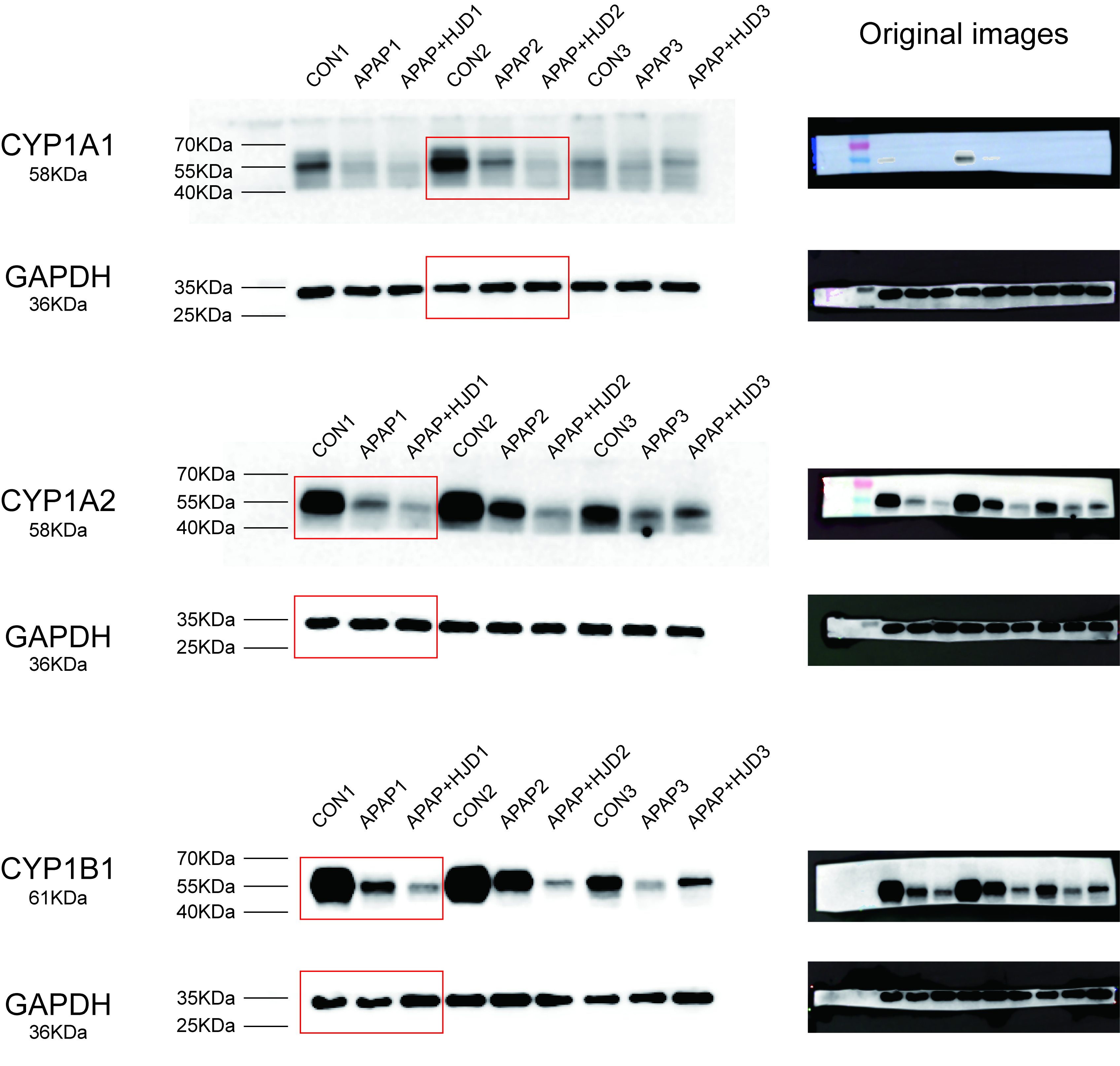

Supplement: Supplementary file 1 — Supplementary Material 1 [file 12906_2024_4517_MOESM1_ESM.tif]
